# Holocene palaeoclimate

**DOI:** 10.1038/s41598-024-75672-y

**Published:** 2024-10-17

**Authors:** Niklas Hausmann, Yoshiki Saito

**Affiliations:** 1 Leibniz Zentrum für Archäologie (LEIZA), Ludwig-Lindenschmit Forum 1, 55116 Mainz, Germany; 2 Estuary Research Center (EsReC), Shimane University, 1060, Nishikawatsu-cho, Matsue, 690-8504 Japan

**Keywords:** Palaeoclimate, Climate sciences

## Abstract

This editorial introduces the “Holocene palaeoclimate” special collection, which examines the intricate relationship between human activities and climate systems throughout the Holocene. The collection highlights the significance of palaeoclimatic reconstructions, providing insights into past climate variability and extremes. By integrating multidisciplinary research from diverse regions, including the Siberian Arctic, Singapore, the Iberian Peninsula, Bavaria, and Madagascar, the collection elucidates the global and regional climate dynamics that shaped historical and contemporary environments. These studies underscore the value of understanding past climates to better predict future climate behaviour and develop strategies to mitigate the impacts of ongoing global warming. The findings offer a long-term perspective on climate trends, contributing to more informed and resilient approaches to addressing contemporary climate challenges.

## Introduction

Contemporary climate research increasingly aims at understanding the intricate but crucial relationship between human activities and climate systems. Palaeoclimatic reconstructions provide invaluable insights into how past societies influenced and were influenced by their environmental contexts, highlighting an interdisciplinary approach that bridges palaeoclimatology, archaeology, and historical ecology^1^. Moreover, our current global challenges require a deeper understanding of this multidirectional relationship between human societies and their surrounding environments, because through them we can better grasp the persistently evolving and multiscalar nature of human influence on climate systems ^2–4^.

Framing these human-climate interactions, periods of significant climate variability and extremes as they are discussed in this Collection are essential to our understanding of historical and contemporary dynamics. Events like the Younger Dryas cold reversal and various Holocene climatic oscillations represent critical junctures in Earth’s climate history^5^. These periods, characterised by rapid and often severe climate shifts, provide crucial data on the mechanisms driving climate change. The study of these episodes in particular can identify the key patterns and triggers that explain similar phenomena in the present day. The Holocene period is riddled with such episodes and the ideal time frame to emphasise the relevance of this information to contemporary climate issues and for predicting and preparing for future climate extremes^6^, which are expected to become more frequent and intense due to ongoing global warming^7^.

At the core of this research lie the results of palaeoclimate reconstructions as presented in this collection. These studies, which make use of isotopic analysis, sediment records, and fossilised materials,, among others, are indispensable for understanding multi-scalar climate variability. They offer a baseline against which modern climate changes can be measured and interpreted, and reveal the complexity and variability of historical climates, offering critical insights into the behaviour of the natural climate system across the Holocene.

## The collection

By integrating data from diverse regions such as the Siberian Arctic, Singapore, the Iberian Peninsula, Bavaria, and Madagascar, this collection enhances our understanding of global climate dynamics. Each regional study contributes unique insights, reflecting local climate patterns and their interactions with broader climatic systems. This comprehensive approach underscores the value of multi-disciplinary and multi-regional research in building a comprehensive understanding of the climatic history of our planet. For instance, Gázquez-Sánchez et al.^8^ investigate the impact of Roman water management on the hydrology of Iberian wetlands using stable isotope analysis of gypsum sediments from Lake Zóñar. The study found that lake levels were significantly lower during Roman times, coinciding with a period of arid climate conditions and increased water diversion to support Roman settlements. This research illustrates the interplay between human activity and natural climate variability in shaping historical hydrological regimes.

A study analysing δ^2^H and δ^18^O isotopes from sediments in Bichlersee, Bavaria, by Prochnow et al.^9^, provided insights into summer palaeohydrology during the Late Glacial and Early Holocene. The isotope records revealed that the Younger Dryas period, although a significant winter phenomenon, had a less pronounced impact on summer climate. By coupling δ^2^H and δ^18^O data, the authors disentangled changes in precipitation isotopic composition from evaporative enrichment, offering a nuanced understanding of past hydrological conditions. These reconstructions offer a baseline against which modern climate changes can be measured and interpreted.

Modelling the past climates, Velay-Vitow et al.^10^ explored the Younger Dryas cold reversal, a period of rapid cooling during the last deglaciation. By applying various freshwater forcings to simulate conditions leading up to the Younger Dryas, the study demonstrated how the collapse and subsequent recovery of the Atlantic Meridional Overturning Circulation influenced Northern Hemisphere temperatures. The research provided key insights into the mechanisms driving rapid climate shifts and the long-term impacts on sea ice and temperature patterns. Understanding these periods is essential for grasping the mechanisms behind rapid climate shifts and their long-term effects on ecosystems and human societies.

In another study, Schubert et al.^11^ reconstructed past climate conditions in the Siberian Arctic using carbon and oxygen isotopes from mummified wood. This innovative approach allowed researchers to estimate precipitation and temperature changes from 3000 years ago. The study revealed that the region experienced increased year-round precipitation and warmer winter temperatures, despite cooler summers compared to today. These findings underscore the complexity of Arctic climate dynamics and provide a historical context for contemporary climate trends. These palaeoclimate reconstructions are indispensable for understanding long-term climate variability.

Research on late Holocene relative sea-level changes by Tan et al.^12^ utilised coral microatolls as indicators. This study produced a precise relative sea-level reconstruction from Singapore, demonstrating the utility of *Diploastrea heliopora* microatolls. The data showed a gradual relative sea-level fall over the past 2800 years, with fluctuations indicating periods of stability and rise. These findings align with regional relative sea-level data and highlight the need for more high-quality records to better understand the drivers behind those changes and refine glacial isostatic adjustment models.

A long-term speleothem record from Anjohibe cave in northwestern Madagascar by Dawson et al.^13^ shed light on Holocene precipitation changes. The study found that precipitation patterns in Madagascar were in phase with the Northern Hemisphere Asian monsoon on millennial scales but exhibited antiphase signals during certain centennial-scale events. The primary driver of these precipitation changes was identified as zonal sea surface temperature gradients in the Indian Ocean, rather than meridional monsoon migrations. This research highlights the complex interactions between regional and global climate systems. Each regional study contributes unique insights, reflecting local climate patterns and their interactions with broader climatic systems.

## Summary

The findings from these palaeoclimate studies have profound implications for future climate predictions and strategies. Many of them provide a human context and help to frame current climate trends within a long-term perspective. Understanding past climate variability and extremes allows scientists to better predict future climate behaviour, especially in terms of frequency and intensity of extreme events. This addition to historical knowledge is crucial for developing robust strategies to mitigate and adapt to ongoing climate changes.

As we face increasing challenges from global warming, insights from palaeoclimate research become ever more critical. They offer a blueprint for understanding how natural systems respond to climatic shifts and human interventions. By acquiring context from the past, we can more effectively ensure that our strategies are informed, resilient, and capable of mitigating the adverse impacts of climate change.
